# Total Plasma Exchange in Hypertriglyceridemia-Induced Pancreatitis: Case Report and Literature Review

**DOI:** 10.1155/2018/4017573

**Published:** 2018-03-04

**Authors:** Harleen Dehal, Michael Adashek

**Affiliations:** Department of Internal Medicine, Sinai Hospital, Baltimore, MD, USA

## Abstract

**Objective:**

To emphasize the role of apheresis in management of pancreatitis.

**Methods:**

The clinical course of a patient admitted for hypertriglyceridemia-induced pancreatitis (HTGP) complicated by multiorgan dysfunction is described, who demonstrated dramatic improvement in his clinical status after total plasma exchange (TPE). In addition, the current guidelines for TPE and the alternative treatment options for HTGP are also presented.

**Results:**

A patient presenting with pancreatitis associated with severe systemic inflammatory response was admitted to our hospital with an initial triglyceride level of 1181 mg/dL. Given the patient's worsening clinical condition, he was started on TPE with a rapid fall in his serum TG levels, in turn leading to early clinical recovery.

**Conclusion:**

Though various therapeutic options for the treatment of HTGP are described in literature, there are no set guidelines available to tackle this difficult clinical situation. TPE, albeit not very well known in this context, is one of the many therapies available. Though it leads to a rapid, precipitous fall in the TG levels and early symptom resolution, the data about the long-term morbidity as well as the effectiveness of this therapy is still lacking.

## 1. Introduction

Pancreatitis in the face of severely elevated triglyceride levels is often encountered in the clinical setting. Though various modalities exist for the management of such patients, apheresis is one such lesser-known therapeutic option. Here, we present a case report and review regarding the current guidelines for initiation of therapeutic plasma exchange in this cohort of patients.

## 2. Case Report

A 52-year-old man with a medical history significant for hypertension and newly diagnosed diabetes initially presented at another facility with a complaint of chest pain and shortness of breath. During a focused cardiopulmonary workup, the patient was incidentally found to have an elevated lipase level of 4900 units/L. Further questioning revealed an extensive history of alcohol abuse, between 56 and 84 grams of alcohol daily for 8 years with last drink < 24 hours prior. An abdominal CT scan revealed pancreatic edema without necrosis, cholelithiasis, choledocholithiasis, or gall bladder thickening. Subsequently, aggressive hydration with lactated Ringer's solution and a broad pancreatitis workup was begun.

The patient's clinical status deteriorated. He became anuric and required intubation for acute respiratory distress syndrome (ARDS) and uremic encephalopathy. The patient was then transferred to our facility. Although there was no history of personal or familial hyperlipidemia, on hospital admission the patient's labs were significant for hypertriglyceridemia of 1181 mg/dL, as well as an ionized hypocalcemia of 0.71 mmol/L and an elevated lipase of 1016 units/L. Fenofibrate was held due to patient intubation; however, a clinical literature review was done showing multiple small studies demonstrating the benefit of early plasmapheresis in this clinical scenario. A central venous catheter was inserted and TPE was begun within 24 hours of admission.

Following the first plasma exchange, the patient's triglycerides (TGs) decreased > 40% to 510 mg/dL. The second session decreased TG to 454 mg/dL. Apheresis was held for one day, during which TG increased to 524 mg/dL at which time apheresis was begun again. A threshold was set for apheresis if TGs were found to be >500 mg/dL, but further apheresis was not required. Following this, the patient was started on a fibrate and daily triglyceride trend was monitored. The patient's ARDS and uremic encephalopathy resolved, and he recovered well from his multiorgan dysfunction state. The patient was discharged to a subacute rehab facility with a TG level of 339 mg/dL.

## 3. Discussion

According to the data from National Health and Nutrition Examination Survey in the United States, between 2009 and 2012, 25.1% of adults over the age of 20 were diagnosed with hypertriglyceridemia (HTG), defined as triglycerides > 150 mg/dL [1]. Hypertriglyceridemia (HTG) is well known to be associated with coronary vascular disease, but data has shown that it plays an important part in stratifying pancreatitis risk according to the Endocrine Society's 2010 classifications of HTG greater than 1000 mg/dL [2].

Elevated triglycerides can be seen in patients with underlying genetic predisposition to abnormal lipoprotein metabolism, compounded by secondary factors such as excessive alcohol intake, insulin resistance, metabolic syndrome, renal or liver diseases affecting TG clearance, and autoimmune disorders, as well as drugs such as protease inhibitors, beta-blockers, and thiazide diuretics. Rarely, a patient with familial HTG without secondary risk factors may also be encountered in clinical practice [3]. These etiologies account for 1–4% of the cases of acute pancreatitis [4].

The mechanism of HTGP (hypertriglyceridemia-induced pancreatitis) is thought to involve increased lipolysis of triglyceride-rich lipid particles by pancreatic lipase, with a resultant production of free fatty acids (FFAs). These FFAs inflict free radical damage to the pancreatic acinar cells, hence initiating a self-perpetuating cycle of pancreatic inflammation and destruction [5]. Although no clear cutoff has been established, it has been observed that the risk for precipitating pancreatitis increases with worsening HTG and is rarely observed with TG < 500 mg/dL [6]. Progressively higher TG levels have been associated with disease severity and multiorgan failure [7].

Treatment of HTGP involves both the conventional pancreatitis treatment of fluid resuscitation and pancreatic rest and also requires a multimodal approach to lower TG levels. Three separate classes of drugs have been shown to be effective in treating hypertriglyceridemia: fibrates decrease triglycerides by 30–50%, niacin by 10–30%, and *n*-3 fatty acids by 20–50%. Statin monotherapy is not recommended in HTG but can be used for dyslipidemia [2]. Other therapies with demonstrated clinical benefit but that have not found a strong foothold in official ACG guidelines for HTGP include plasmapheresis, insulin monotherapy, or insulin/heparin dual therapy.

Insulin potentiates lipoprotein lipase's (LPL) ability to degrade chylomicrons that house triglycerides, whereas heparin stimulates release of LPL from the endothelial membrane. Subsequently, both heparin and insulin have a similar mechanism in increasing the role of LPL and decreasing serum triglycerides [8].

Apheresis broadly describes the removal of a blood component or components. Therapeutic plasma exchange (TPE) involves removal of patient plasma and replacement with colloid or crystalloid solutions. TPE has proven beneficial for HTGP in various small studies and a few case reports and currently has a Category III indication [9]. TPE can cause a rapid decline in the TG levels, with a documented decrease by as much as 70% after a single session [10]. Although there are no set guidelines for timing of initiation of TPE, there is some evidence from small studies and case reports that early initiation of plasmapheresis within 48 hours of presentation results in improved morbidity and mortality. TPE should be considered in patients with lactic acidosis or evidence of end organ dysfunction [11, 12]. Current data regarding the replacement fluid in TPE is sparse; historically, both albumin and fresh frozen plasma (FFP) have been used. FFP has a theoretical advantage over albumin in that it provides the patient with lipoprotein lipase and apolipoprotein, both necessary for the hydrolysis of TGs [13]. Patients may need multiple cycles of TPE until the TG levels fall below 500 mg/dL.

Although TPE results in rapid lowering of TG, it is not primary therapy to maintain HTGP remission. To prevent HTGP recurrence, lifestyle modification including low-fat diet, alcohol cessation, and exercise as well as medications such as fibrates, niacin, and *n*-3 fatty acids are the cornerstone of therapy. Patients with hyperlipidemia may fail to achieve adequate TG control < 500 mg/dL to prevent repeat episodes of pancreatitis and may be good candidates for intermittent TPE treatment [14].

TPE results in early symptom resolution; however, the data about the long-term morbidity as well as the effectiveness of this therapy versus standard therapy is lacking. Large-scale studies are needed to further assess the efficacy of TPE as well as to develop guidelines about the adequate timing for commencing therapy.
